# Evidence of Increase in Mortality After the Introduction of Diphtheria–Tetanus–Pertussis Vaccine to Children Aged 6–35 Months in Guinea-Bissau: A Time for Reflection?

**DOI:** 10.3389/fpubh.2018.00079

**Published:** 2018-03-19

**Authors:** Peter Aaby, Søren Wengel Mogensen, Amabelia Rodrigues, Christine S. Benn

**Affiliations:** ^1^Bandim Health Project, Indepth Network, Bissau, Guinea-Bissau; ^2^Research Centre for Vitamins and Vaccines (CVIVA), Bandim Health Project, Statens Serum Institut, Copenhagen, Denmark; ^3^OPEN, Institute of Clinical Research, University of Southern Denmark, Odense University Hospital, Odense, Denmark

**Keywords:** bias in vaccine studies, diphtheria–tetanus–pertussis vaccine, heterologous effects, measles vaccine, non-specific effects of vaccines, oral polio vaccine

## Abstract

**Background:**

Whole-cell diphtheria–tetanus–pertussis (DTP) and oral polio vaccine (OPV) were introduced to children in Guinea-Bissau in 1981. We previously reported that DTP in the target age group from 3 to 5 months of age was associated with higher overall mortality. DTP and OPV were also given to older children and in this study we tested the effect on mortality in children aged 6–35 months.

**Methods:**

In the 1980s, the suburb Bandim in the capital of Guinea-Bissau was followed with demographic surveillance and tri-monthly weighing sessions for children under 3 years of age. From June 1981, routine vaccinations were offered at the weighing sessions. We calculated mortality hazard ratio (HR) for DTP-vaccinated and DTP-unvaccinated children aged 6–35 months using Cox proportional hazard models. Including this study, the introduction of DTP vaccine and child mortality has been studied in three studies; we made a meta-estimate of these studies.

**Results:**

At the first weighing session after the introduction of vaccines, 6–35-month-old children who received DTP vaccination had better weight-for-age *z*-scores (WAZ) than children who did not receive DTP; one unit increase in WAZ was associated with an odds ratio of 1.32 (95% CI = 1.13–1.55) for receiving DTP vaccination. Though lower mortality compared with not being DTP-vaccinated was, therefore, expected, DTP vaccination was associated with a non-significant trend in the opposite direction, the HR being 2.22 (0.82–6.04) adjusted for WAZ. In a sensitivity analysis, including all children weighed at least once before the vaccination program started, DTP (±OPV) as the most recent vaccination compared with live vaccines or no vaccine was associated with a HR of 1.89 (1.00–3.55). In the three studies of the introduction of DTP in rural and urban Guinea-Bissau, DTP-vaccinated children had an HR of 2.14 (1.42–3.23) compared to DTP-unvaccinated children; this effect was separately significant for girls [HR = 2.60 (1.57–4.32)], but not for boys [HR = 1.71 (0.99–2.93)] (test for interaction *p* = 0.27).

**Conclusion:**

Although having better nutritional status and being protected against three infections, 6–35 months old DTP-vaccinated children tended to have higher mortality than DTP-unvaccinated children. All studies of the introduction of DTP have found increased overall mortality.

## Key Observations

• DTP and oral polio vaccine (OPV) were first introduced to children aged 6–35 months in June 1981 in an urban area in Guinea-Bissau. Children who were DTP-vaccinated at the first weighing session after the introduction of DTP had significantly better weight-for-age *z*-scores than those not vaccinated.
• Although better survival was expected, the DTP-vaccinated children had twofold higher mortality than DTP-unvaccinated children.
• In a meta-analysis of the three studies of the introduction of DTP in urban and rural Guinea-Bissau, DTP-vaccinated children had twofold higher mortality than DTP-unvaccinated children.

## Introduction

Whole-cell diphtheria–tetanus–pertussis (DTP) vaccine is the most commonly used vaccine in low-income countries with poor health infrastructure, and the coverage for the third dose of DTP-containing vaccines (DTP3) is the main performance indicator for vaccination programs (1). However, no prospective study has shown that receiving DTP is associated with better child survival (2, 3). On the contrary, in the past 20 years several studies have suggested that DTP is associated with increased child mortality, particularly for girls (2–4).

We recently examined what happened when DTP and oral polio vaccine (OPV) were introduced to infants aged 3–5 months in Guinea-Bissau in June 1981 in connection with tri-monthly weighing sessions in an urban community in Bandim (5). In this age group, the child’s date of birth determined whether a child was vaccinated early or late. Children who were just over 3 months old at the time of the tri-monthly weighing sessions were vaccinated at that age; those who were just below 3 months old would only be vaccinated for the first time at almost 6 months of age. In this “natural experiment,” DTP-vaccinated children had fivefold higher mortality between 3 and 6 months of age than children not yet vaccinated with DTP (5).

When we initiated vaccination with DTP and OPV in Guinea-Bissau in June 1981, we also offered a catch-up program to children aged 6–35 months attending the weighing sessions. This situation provides an opportunity to compare the survival of older DTP-vaccinated and DTP-unvaccinated children.

In principle, children above 3 months of age attending the weighing sessions were offered vaccination if vaccines and equipment (syringes, sterilization stove) were available. However, nurses and mothers were reluctant to vaccinate sick or weak children. Other reasons for not being vaccinated were that the children were temporarily traveling, or that they stayed for prolonged periods in the rural areas where access to health care was limited and child mortality was higher. Thus, apart from the specific disease-protective effect of DTP, inherent biases would lead one to expect that DTP-vaccinated children had better survival than DTP-unvaccinated children.

## Materials and Methods

### Background

Bandim Health Project (BHP) has followed an urban community in the capital of Guinea-Bissau with a demographic surveillance system since December 1978. The national immunization program in Guinea-Bissau started in 1986 with funding from UNICEF. From January 1980, BHP conducted tri-monthly weighing sessions of all children in the community to identify malnourished children. From June 1981, vaccinations were offered in connection with these weighing sessions.

### Demographic Surveillance

When the project started in 1978, child mortality was very high. Malnutrition was assumed to be the main cause and a study was, therefore, initiated to determine why children were malnourished (6–8). The area was mapped and a census was conducted (5). Four female health workers identified pregnant women, encouraged women to attend the antenatal clinic in the study area, and followed children with anthropometric measurements to assess growth patterns and detect malnourished children. Each health worker followed a population of 1,500–2,000 individuals, the total number of individuals in Bandim being around 6,300 at the beginning of the study. The health worker kept a list of children under 3 years of age in each of the eight sub-districts in Bandim. An expatriate nurse supervised the health workers. BHP had no computerized surveillance system when the study started, but BHP kept an A5 card (“BHP card”) for each child, where weights and vaccination dates were noted. With a birth rate around 5%, the annual birth cohort was around 300–350 newborns.

The Bandim population was very mobile for many reasons. First, it was important to maintain contact with the natal village for ceremonial purposes and to secure access to rice, often by helping the family during the rice production cycle. Second, many women tried to obtain cash income by growing fruits or vegetables in the rural areas or by producing cashew wine to be sold in Bissau. Third, mothers were not supposed to have sexual relations during breastfeeding as semen is believed to damage breastmilk causing diarrhea in the child (9). Breastfeeding was prolonged in Guinea-Bissau, between 18 and 36 months in different ethnic groups. Thus, many women preferred to stay in the rural areas with their family while breastfeeding. These cultural patterns meant that some mothers and children were away for long periods. Typically, there would be family members in Bandim, who we could ask about the whereabouts of the child.

### Tri-Monthly Weighing Sessions

We arranged tri-monthly weighing sessions in each sub-district (8). The health worker in charge advised mothers the day before a session. If a child was not present, its vital status was ascertained by asking the family. The following morning, the child’s weight was measured on a hanging Salter scale and noted on the child’s health card and the BHP card.

### Vaccinations

There was no community vaccination program in Guinea-Bissau when BHP started vaccinations. Mother could have taken their children to the clinic of the Mother and Child Health Program in town. This clinic was mainly attended by the urban elite so very few children from Bandim had received routine vaccinations (5). In June 1981, BHP started to provide vaccinations at the tri-monthly weighing sessions. A health center nurse accompanied the nutrition team and vaccinated eligible children.

Eligible children were between 3 months and 3 years of the age. However, some children in this age group were not vaccinated. Both nurses and mothers thought that sick or otherwise weak children should not be vaccinated. The BHP card often indicated that the child was “sick,” “malnourished,” or “orphan” as an explanation of why an age-eligible child had not been vaccinated. Other reasons for not vaccinating an age-eligible child were temporary shortages of vaccines or syringes.

**Figure 1:**
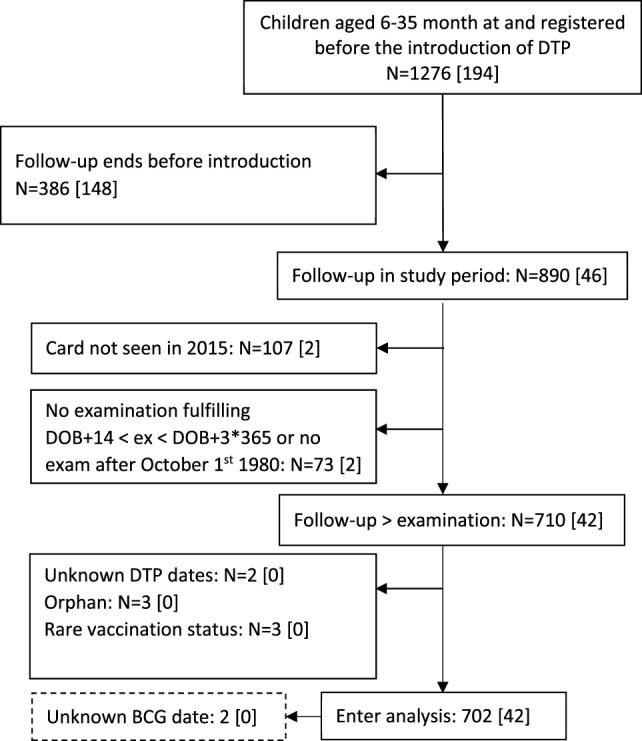
Flowchart of study population and children included in the analyses. DOB, date of birth; [], died during follow-up. Children were only included in the main analysis if they had taken part in a weighing session after October 1, 1980.

The three DTP and OPV doses could be given from 3 months of age with an interval of 1 month, but since we only performed weighing sessions every 3 months, most children had longer intervals between the three doses. Also, there were several periods where either OPV or DTP was missing [Ref. (5), Figure 1]. The expatriate nurse sometimes organized additional vaccination sessions in which the children were not weighed, but vaccinations were noted on the BHP cards.

### Data Control

Weights and vaccinations from the BHP cards were entered into a computerized system in 1990–1991. For the present analysis, information on dates of visit, weights, and vaccination dates was checked against the original cards in 2015.

### The Study Cohort and Vaccination Analyses

We included children born between June 1978 and December 1980 and hence aged 6 and 35 months in June 1981 when DTP and OPV vaccines became available (Figure 1). Furthermore, it was an inclusion criterion that children were registered in the area before the vaccinations started. We excluded orphans, since they were not breastfed and were likely to have different care; their mortality was very high (10). Children who never attended a weighing session after birth registration were not included in the analysis, since their mothers had likely left for the rural areas. In the analyses, we restricted the data set to children taking part in at least one weighing session after October 1980, 8–9 months before vaccinations started. This was done to assure that the children had been seen fairly recently and were, therefore, likely to be around when the vaccinations started. Since the children were called every 3 months, the time of death or migration out of the area is fairly accurate.

### Vaccination Analyses

We conducted three complementary analyses to assess the effect of DTP on child survival.

#### Analysis 1

We compared DTP-vaccinated children and those who were not DTP-vaccinated when they came for their first weighing session after the introduction of vaccinations in June 1981. Since not all children were included, the analysis had less power. We followed children from their first weighing session and until they received their next vaccination or they migrated, died, or turned 3 years of age. Thus, children had to be present at a weighing session to be included in this analysis and we could adjust for the weight-for-age *z*-score (WAZ) obtained at that session.

#### Analysis 2

**Figure 2:**
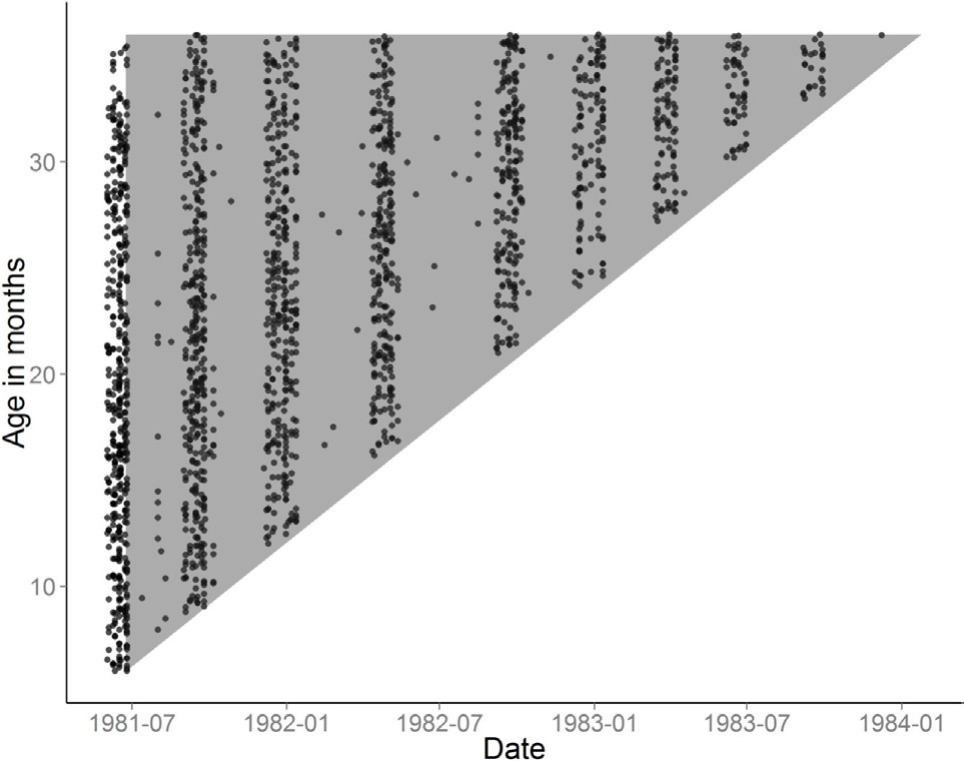
Examinations are plotted on the axes of age and calendar time. Each dot corresponds to a weighing examination of a child. The gray triangle illustrates the age groups and periods, where follow-up time was included in the survival analysis. The approximate tri-monthly regime of examinations is visible in the distribution of dots on the horizontal axis.

In this analysis, children were considered DTP-vaccinated from the date they received their first DTP vaccination (with or without OPV) in June 1981, or at one of the subsequent weighing sessions (Figure 2). Children were considered DTP-unvaccinated from the date vaccination was first offered in their sub-district, irrespective of whether they were present at the weighing session, and until they were DTP-vaccinated at a subsequent session, migrated, died, or turned 3 years of age. (The difference between this analysis and Analysis 1 was that children were considered DTP-unvaccinated if they were age-eligible, irrespective of whether they had attended a weighing session or not, and vaccination status could change during follow-up, so a child could contribute risk time first as DTP-unvaccinated and then as DTP-vaccinated.)

#### Analysis 3

In the third analysis, we compared mortality of children according to their most recent vaccination status; DTP-vaccinated children were compared with children who had received no vaccination or live vaccine only (MV, OPV, or MV + OPV) as their most recent vaccination.

### Statistical Analyses

The survival of different vaccination groups was compared using a Cox proportional hazard model with age as underlying time. Thus, age was inherently controlled in this analysis. The WHO WAZ was used to assess nutritional status. In analysis 1 in which we compared children who had attended weighing sessions and been vaccinated or not vaccinated we adjusted the analysis for nutritional status (WAZ score). Since we provided almost all vaccines, most vaccinations were known from the date of vaccination, but a few children were vaccinated elsewhere. To avoid survival bias, we used a landmark approach in all analyses (11); hence, a child’s vaccination status was only updated from the day the information was collected.

### Studies of the Introduction of DTP

Including this study, there are only three studies of the introduction of DTP, all from Guinea-Bissau (5, 12). We made a meta-estimate for these studies, since they represent an unusual set of circumstances in relation to the discussion of potential biases in studies of the non-specific effects of vaccines (13–17). First, in all three studies the nutritional status was worse for children not vaccinated. Second, we administered nearly all vaccines, so most dates of vaccination were known precisely. Third, there were no campaigns with other vaccines or micronutrient supplements at the time of these studies. Fourth, they represent all the data sets available on the introduction of DTP in Guinea-Bissau, so reporting bias is not an issue (15).

## Results

Of the 890 children aged 6–35 months registered in Bandim in June 1981, we were not able to locate the BHP card of 107 (12%) children in 2015; most will not have attended an examination, but some cards may have been lost. A further 81 had a BHP card, but had not attended a weighing session since October 1980, had no precise vaccination dates, or were excluded due to other considerations (see Figure 1). Hence, 702 children were included in the study cohort; the number of deaths and person-years in the different vaccine groups was, therefore, limited (Table S1 in Supplementary Material).

The temporal distribution of weighing sessions in this cohort is shown in Figure 2. As documented in Table S2 in Supplementary Material, 82 and 84% received DTP1 and OPV1 before they reached 3 years of age, the median ages of vaccination being 633 and 614 days, respectively. It should be noted that only 38 and 49% of the children received all three doses of DTP and OPV, respectively, before 3 years of age. Due to earlier MV campaigns (6, 7), 82% had received MV at a median age of 348 days. There were 42 deaths between 6 and 35 months of age; 14 had fever as the main symptom, 13 had diarrhea or diarrhea and vomiting, 6 died from measles, 1 had respiratory infection, 1 was malnourished, 1 had anemia, 1 did not eat, and 5 had no information, most likely because the mother/family had moved.

We compared background factors for DTP-vaccinated children and children who remained DTP-unvaccinated until at least 3 years of age (Table 1). The DTP-vaccinated children were far more likely to have attended weighing sessions, to have received measles vaccine (MV) in the campaigns, or to have received DTP at the Mother and Child Clinic before June 1981 (6, 7). There were no differences in distribution of the sexes, twins, or ethnic groups.

**Table 1:** Background factors for 6–35 months old children who were vaccinated or not vaccinated at their first weighing session in June 1981.

Analysis 1	Diphtheria–tetanus–pertussis (DTP)-vaccinated at or before first session	DTP-unvaccinated in first session
Mean weight-for-age *z*-score (SD) at first examination	−0.83 (0.06) [394]^¤^	−1.17 (0.08) [197]^¤^

**Analysis 2**	**DTP-vaccinated during follow-up**	**Not DTP-vaccinated during follow-up**

*N*	553	149
Male sex	51% (282)	53% (78)
Twin	3% (15)	2% (3)
Ethnic group		
Pepel	51% (282)	48% (71)
Balanta	15% (84)	17% (25)
Other ethnic groups	34% (187)	36% (53)
Measles vaccinated before June 1981	71% (391)	58% (86)
DTP before June 1981	6% (32)	
Classified as malnourished	6% (33)	5% (8)
Mean number (SD) of weighing sessions per year after start of vaccinations	2.57 (0.06)^#^	0.91 (0.09)^#^

*^#^Comparison p < 0.0001*.

*^¤^Comparison p = 0.001*.

### Analysis 1

At the first weighing session after the vaccinations started in June 1981, the WAZ was much higher for the children who received DTP (WAZ −0.83) than for those children who did not receive DTP (WAZ −1.17) (Table 1). An increase of one *z*-score was associated with an odds ratio (OR) of 1.32 (95% CI = 1.13–1.55) for being vaccinated at the first weighing session. Compared with not being DTP-vaccinated, DTP vaccination at the first weighing session was associated with a non-significant mortality hazard ratio (aHR) of 2.22 (95% CI = 0.82–6.04) adjusted for WAZ (Table 2), the aHR being 7.03 (0.88–56.04) for girls, and 1.28 (0.38–4.25) for boys (test for interaction *p* = 0.17).

**Table 2:** Analysis 1: mortality rates (MR) per 100 person-years and hazard ratios (HR) of 6–35 months old children who were either diphtheria–tetanus–pertussis (DTP)-vaccinated or not DTP-vaccinated at their first examination.

	Mortality rate (deaths/person-years)		HR (95% CI)	HR (95% CI), adjusted for weight-for-age*z*-scores (WAZ)
			
Vaccination status	DTP (±OPV)	No DTP
All children	9.68 (18/185.9) [394]	4.80 (5/104.1) [197]	2.01 (0.74–5.41)	2.22 (0.82–6.04)
Girls	11.15 (9/80.7) [191]	1.86 (1/53.7) [100]	6.67 (0.84–52.84)	7.03 (0.88–56.04)
Boys	8.58 (9/104.9) [202]	8.07 (4/49.6) [96]	1.04 (0.32–3.40)	1.28 (0.38–4.25)

*Bandim, 1981–1983*.

*Children who have received DTP before June 2, 1981 were censored from the analysis*.

### Analysis 2

Including all children in the cohort, following them to 3 years of age and allowing children to change status during follow-up when new information was collected at a weighing session, having received DTP was associated with a non-significant HR of 1.48 (0.72–3.06) (Table 3). The HR was 2.91 (0.84–10.00) for girls and 0.88 (0.34–2.62) for boys.

**Table 3:** Analysis 2: mortality rates (MR) per 100 person-years and hazard ratios (HR) of 6–35 months old DTP-vaccinated and diphtheria–tetanus–pertussis (DTP)-unvaccinated children.

	**Mortality rate (deaths/person-years)**		**HR (95% CI)**
		
	**DTP(±OPV)**	**No DTP**	
All	5.4 (32/590.6) [553]	4.1 (10/242.5) [327]	1.48 (0.72–3.06)
Girls	6.9 (19/273.5) [270]	2.6 (3/116.4) [155]	2.91 (0.84–10.00)
Boys	4.1 (13/316.5) [282]	5.6 (7/125.1) [170]	0.88 (0.34–2.62)

*Bandim, 1981–1983*.

*178 children were first DTP-unvaccinated and then received DTP during follow-up. Three children had no information on sex. If we adjusted for the most recent WAZ measurement, the HR of 1.48 (0.72–3.06) became 1.52 (0.74–3.15)*.

### Analysis 3

Children who received DTP (with or without OPV) as the most recent vaccination had an HR of 1.77 (0.93–3.38) compared with children who had received a live vaccine or no vaccine at all and had a HR of 1.90 (0.92–3.94) if compared only with children who had received live vaccine only (Table 4). In a sensitivity analysis, including also the 47 children whose most recent weighing session had been before October 1980, the HRs for DTP was 1.89 (1.00–3.55) (Table 4), the HR being 2.76 (1.07–7.07) for girls, and 1.34 (0.56–3.22) for boys.

**Table 4:** Analysis 3: mortality rates (MR) per 100 person-years and hazard ratios (HR) of 6–35 months old children in relation to most recent vaccination.

	Mortality rate (deaths/person-years)		HR (95% CI)Had weighing session after October 1, 1980^a^	HR (95% CI) All children^b^
			
Vaccination status	Diphtheria–tetanus–pertussis (DTP) (±OPV)	No DTP
All	6.2 (28/451.0) [535]	3.7 (14/382.0) [539]	1.77 (0.93–3.38)	1.89 (1.00–3.55)
Vaccination status	DTP(±OPV)	Only live vaccine (MV, OPV, or MV + OPV)		
All	6.2 (28/451.0) [535]	3.3 (10/303.84) [473]	1.90 (0.92–3.94)	1.99 (0.96–4.12)

*Bandim, 1981–1983*.

*^a^See Figure 1; Adjustment for the most recent weight-for-age z-scores measurement did not change the estimate*.

*^b^Include 47 children whose most recent weighing session prior to June 1981 had been before October 1980*.

Though the group was small, it is worth noting that children who received OPV-only had low mortality (Table S1 in Supplementary Material), the HR for DTP(±OPV)-vaccinated compared with OPV-only vaccinated children was 3.76 (0.89–15.83).

### Studies of the Introduction of DTP

**Figure 3:**
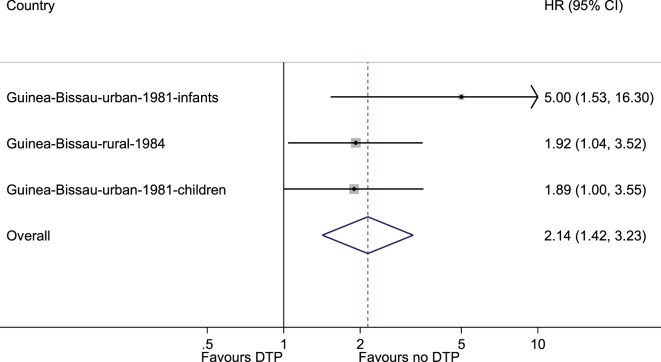
Meta-analysis of the three studies of the introduction of diphtheria–tetanus–pertussis. The fixed effects model gave an estimate of 2.14 (1.42–3.23) and the random effects model gave 2.17 (1.39–3.38).

In the three studies of introduction of DTP in rural and urban Guinea-Bissau, DTP vaccination was associated with a HR of 2.14 (1.42–3.23) compared with DTP-unvaccinated children (Figure 3). The negative effect was separately significant for girls [HR = 2.60 (1.57–4.32)], but not for boys [HR = 1.71 (0.99–2.93)] (Table S3 in Supplementary Material) (test for interaction *p* = 0.27).

## Discussion

Although lower mortality was expected for DTP-vaccinated children compared with the frail unvaccinated children, DTP vaccination was associated with higher mortality, particularly in girls.

### Strength and Weaknesses

The home-visits preceding each of the tri-monthly weighing sessions ensured that we had follow-up information for all children and relatively accurate information on the time of movement or death. In the initial analyses, we included only children who had attended the 3-monthly weighing sessions at least once within the last 8–9 months (5). This meant that children mostly living outside the area were not included; these children had no access to community vaccinations and they lived elsewhere where the mortality risk may well have been much higher. We had to exclude some children because their card could not be found (Figure 1). The excluded groups (Figure 1) did not have high mortality so the increased mortality of DTP-vaccinated children is not due to exclusion of unvaccinated children with a particularly high mortality. When we increased the power of the study by including children only seen before October 1980 (Figure 1), the HR estimate for DTP-vaccinated versus DTP-unvaccinated as most recent vaccine changed from 1.77 (0.93–3.38) to 1.89 (1.00–3.55).

The inherent biases in this study are clearly in favor of the DTP-vaccinated children (2): first, the DTP vaccine protects against three severe diseases. Second, the DTP-unvaccinated children were usually children deemed too sick or too weak to be vaccinated, as evidenced by the nurse’s notes on the BHP card and by the fact that these children had worse nutritional status. Third, DTP-unvaccinated children attended the weighing sessions less frequently (Table 1) and were, therefore, more likely to be staying for longer periods in the rural areas where the mortality risk was higher (12). Noteworthy, we were able to obtain mortality information from these children because their father and other relatives stayed in the study area.

WHO experts have argued that the negative effect of DTP is exaggerated, because studies have only been conducted in situations with herd immunity against pertussis and where the benefit of preventing pertussis would not be seen (13). However, pertussis was endemic in the 1980s before the roll out of the vaccination program in Guinea-Bissau, but all three studies of the introduction of DTP into urban and rural areas of Guinea-Bissau showed excess mortality associated with DTP vaccination (5, 12).

### Comparison With Previous Studies of DTP and OPV

This study was small (Table S1 in Supplementary Material) and many results were not statistically significant; some variability was, therefore, also to be expected. Still the results were very similar to the previous studies of the introduction of DTP and OPV. All three studies have accurate assessment of vaccination status and prospective follow-up; all three studies have found that DTP is associated with an increase in all-cause mortality (3). A previous meta-analysis suggested twofold higher mortality for DTP-vaccinated children (3). However, this is probably an underestimation of the “true” effect since the unvaccinated group is usually affected by various negative health selection biases, including frailty bias. In the best studies, with no selection bias or good control for frailty bias, DTP has been associated with four to five times higher mortality (2, 5).

As in this study, we have previously found excess female mortality after DTP vaccination (4). In our previous meta-analysis, we compared DTP-vaccinated females with DTP-unvaccinated, but BCG-vaccinated females, and DTP was associated with an HR of 2.54 (1.68–3.86). In the three studies of the introduction of DTP very few of the DTP-unvaccinated had received BCG. Hence, DTP seems to have a marked negative effect for females irrespective of whether one compares DTP-vaccinated girls with totally unvaccinated or with BCG-vaccinated girls.

There have been few studies of OPV administered alone (5). It is, therefore, worth noting that the small number of children who received OPV only had lower mortality than DTP-vaccinated children in this study (Table S1 in Supplementary Material), although the difference was not statistically significant. We have recently been able to document marked beneficial effects of OPV on all-cause mortality in both randomized trials and in natural experiments with OPV campaigns (18, 19).

### Interpretation

Various WHO committees have previously reviewed the non-specific effects of vaccines and have dismissed the possibility that DTP could have negative effects, and have suggested that the negative effect of DTP is likely to be explained by uncontrolled confounding or bias (13–17). Recently, the Strategic Advisory Group of Experts on Immunization sponsored a review of the potential non-specific effects of BCG, DTP, and MV (15, 16). Though it was noted that the majority of studies (7/10) showed a deleterious effect of DTP, the evidence was considered inconsistent because two studies showed a beneficial effect. Furthermore, the review invoked “a high risk of bias” for all the observational studies (17).

However, it is important to consider the direction of bias. All documented biases favor the vaccinated group because vaccination is usually delayed in unhealthy children, and DTP-unvaccinated children should, therefore, have a higher mortality than vaccinated children (2, 3). The WHO review mentioned four potential biases, which would favor the unvaccinated group (15). First, sick children might come more often to a health center for consultation and, therefore, be more likely to receive DTP, since WHO has recommended vaccination of sick children; this bias was clearly not relevant in Guinea-Bissau, where neither nurses nor mothers thought that a sick child should be vaccinated. Second, starting follow-up from a survey sometime after the actual DTP vaccinations had been administered, as would often happen in a setting, where vaccination information is collected with intervals, could potentially mean that frail children in the unvaccinated group had already died, and that the DTP-vaccinated children, therefore, had an “unnaturally” high mortality (15). The one study testing this found no evidence for such a bias (20) and more importantly, several studies, including all three studies of the introduction of DTP in Guinea-Bissau, started observation at the date of vaccination for almost all children and found strong negative effects. Hence, this bias was not relevant in the present study. Third, censoring follow-up at subsequent MV would remove some of the best children from the DTP-vaccinated group and, therefore, gives higher mortality in the DTP group (15). Again, the studies that have tested this potential bias have not found evidence for such a bias (21) but, more importantly, several studies—like the present one—did not censor for MV and found equally strong negative effects for DTP (2). Hence, this bias was not relevant in this study. Fourth, it has been discussed whether a bias in reporting could have played a role (15). The observation of increased mortality after DTP was reported more than 15 years ago (22), and has not been contradicted by a properly conducted prospective study. In contrast, several other groups have reported that DTP was associated with increased overall mortality (23–25) or higher female than male mortality (23, 26–28). Hence, reporting bias is a very unlikely explanation. We have now reported all the possible data sets from when DTP was introduced in both urban and rural areas of Guinea-Bissau (5, 12); all showed a negative effect of DTP vaccination. Hence, reporting bias is not relevant in relation to the studies of the introduction of DTP from Guinea-Bissau. Therefore, the three studies of the introduction of DTP from Guinea-Bissau (5, 12) are not affected by the theoretical biases used to recommend caution in the interpretation of observational studies suggesting deleterious effects from DTP (15–17).

The specific immunological mechanisms explaining why DTP and OPV have NSEs have not yet been identified. However, there is an increasing evidence that live vaccines (BCG, Vaccinia) induce innate immune training producing stronger pro-inflammatory responses which may lead to protection against unrelated infections (29, 30). In contrast, studies of non-live vaccine have suggested that they may induce tolerance which could enhance the susceptibility to unrelated infections (31). The pattern of worse effects for females than for males have turned out to be systematic for several non-live vaccines, including DTP (4, 26), inactivated polio vaccine (32), hepatitis B vaccine (33), pentavalent vaccine (34), and RTS,S malaria vaccine (35). This pattern has not been studied from an immunological perspective and an explanation has still to be found.

### Implications and Conclusion

Our data clearly showed that DTP vaccinations were delayed in unhealthy children. Hence, healthier children received DTP first, and DTP-unvaccinated children should, therefore, have had a higher mortality rate. Despite this, DTP was associated with increased child mortality, particularly for girls. All three studies of the introduction of DTP vaccine found negative effects which are different from what should have been expected due to the disease-preventive effects of the vaccine and the inherent biases favoring vaccinated children (5, 12). The results are also in stark contrast to the studies of the introduction of measles vaccine, which uniformly show very strong mortality reductions (6, 7, 15). Hence, the studies of the introduction of DTP constitute a clear danger signal that DTP may substantially increase all-cause mortality.

Adding to the danger signal, DTP is associated with increased female mortality relative to male mortality in all available studies. Girls did not have higher mortality than boys in the pre-vaccination era in West Africa (2). Hence, there is a need for further research to assess the overall mortality effect of DTP and how the negative effects of DTP can be removed or modified. For example, co-administration of BCG and DTP may reduce the negative effect of DTP (21). Randomized trials have also shown that MV or BCG administered shortly after DTP may reduce the negative effect of DTP and lower mortality (2). Such alternative immunization strategies should be further tested in randomized trials.

Given the threat from diphtheria, tetanus, and pertussis and the less-effective acellular pertussis vaccine used in many countries, it is understandable that there has been reluctance in accepting that DTP could have negative effects for overall health in low-income countries. However, the studies from low-income countries have been consistent in showing deleterious effect of DTP (3); furthermore, the first studies are now showing that non-live and live vaccines have differential NSEs on hospital admissions for infectious diseases in high-income countries (36, 37). Hence, it would seem to be high time to settle whether DTP has negative effects on overall child health and if it has negative effects to explore whether alternative vaccination schedules could remove the problem.

In the current global immunization system, the coverage for the third dose of DTP (DTP3) is used as the main performance indicator for national immunization programs. This will clearly lead to an emphasis on increasing the coverage for DTP3 (1) more than the coverage for other vaccines. Given that all studies, including the present one, suggest that DTP is associated with increased female mortality, this is really an illogical position. We need to use program performance indicators which are positively associated with better child survival.

## Independence

The funding agencies had no role in the study design, data collection, data analysis, data interpretation, or the writing of the report.

## Transparency

The first author affirms that the manuscript is an honest, accurate, and transparent account of the study being reported; that no important aspects of the study have been omitted; and that any discrepancies from the study as planned (and, if relevant, registered) have been explained.

## Data Sharing

Through request to the authors.

## Ethics Statement

The study of nutritional status was planned between the SAREC (Swedish Agency for Research Collaboration with Developing Countries) and the Ministry of Health in Guinea-Bissau. There were no ethical committees for approval of health research at the time of the study. The study was explained to the population in community meetings organized by the local committee and the researchers prior to initiation of data collection. Consent was not sought from individual mothers, since the project implemented intended national policies for nutritional surveillance and immunization.

## Author Contributions

CB and PA proposed the study. PA collected the original data. AR is responsible for the demographic surveillance system. SM and PA cleaned the data. SM conducted the statistical analyses. The first draft was written by PA; all authors contributed to the final version of the paper. PA will act as guarantor of the study.

## Conflict of Interest Statement

The authors declare that the research was conducted in the absence of any commercial or financial relationships that could be construed as a potential conflict of interest.

## References

[1] Fisker AB Hornshøj L Rodrigues A Balde I Fernandes M Benn CS Effects of the introduction of new vaccines in Guinea-Bissau on vaccine coverage, vaccine timeliness, and child survival: an observational study. Lancet Glob Health (2014) 2:e478–87. 10.1016/S2214-109X(14)70274-8 25103521

[2] Aaby P Benn CS Nielsen J Lisse IM Rodrigues A Ravn H. Testing the hypothesis that diphtheria-tetanus-pertussis vaccine has negative non-specific and sex-differential effects on child survival in high-mortality countries. BMJ Open (2012) 2:e000707. 10.1136/bmjopen-2011-000707 22619263 PMC3364456

[3] Aaby P Ravn H Benn CS The WHO review of the possible non-specific effects of diphtheria-tetanus-pertussis vaccine. Pediatr Infect Dis J (2016) 35:1247–57. 10.1097/INF.0000000000001269 27753772

[4] Aaby P Ravn H Fisker AB Rodrigues A Benn CS. Is diphtheria-tetanus-pertussis (DTP) associated with increased female mortality? A meta-analysis testing the hypothesis of sex-differential non-specific effects of DTP vaccine. Trans R Soc Trop Med Hyg (2016) 110:570–81. 27856947 10.1093/trstmh/trw073 PMC5155548

[5] Mogensen SW Rodrigues A Fernandes M Benn CS Ravn H Aaby P The introduction of diphtheria-tetanus-pertussis and oral polio vaccines among infants in an urban African community: a natural experiment. EBioMedicine (2017) 17:192–8. 10.1016/j.ebiom.2017.01.041 28188123 PMC5360569

[6] Aaby P Bukh J Lisse IM Smits AJ. Measles vaccination and reduction in child mortality: a community study from Guinea-Bissau. J Infect (1984) 8:13–21. 6699411 10.1016/s0163-4453(84)93192-x

[7] Mogensen SW Aaby P Smedman L Fernandes M Martins CL Rodrigues A The introduction of standard measles vaccination in an urban African community. BMJ Open (2016) 6(12):e011317 10.1136/bmjopen-2016-011317 PMC5223649 27998896

[8] Aaby P Bukh J Lisse IM Smits AJ. Measles mortality, state of nutrition, and family structure: a community study from Guinea-Bissau. J Infect Dis (1983) 147:693–701. 6842007 10.1093/infdis/147.4.693

[9] Jakobsen MS Sodemann S Mølbak K Alvarenga IJ Nielsen J Aaby P. Termination of breastfeeding after 12 months of age due to a new pregnancy and other causes is associated with increased mortality in Guinea-Bissau. Int J Epidemiol (2003) 32:92–6. 10.1093/ije/dyg006 12690017

[10] Masmas T Jensen H da Silva D Co A Høj L Sandström A Survival among motherless children in rural and urban in Guinea-Bissau. Acta Paediatr (2004) 93:99–105. 10.1111/j.1651-2227.2004.tb00682.x 14989448

[11] Jensen H Benn CS Lisse IM Rodrigues A Andersen PK Aaby P Survival bias in observational studies of the impact of routine vaccinations on childhood survival. Trop Med Int Health (2007) 12:5–14. 10.1111/j.1365-3156.2006.01773.x 17207143

[12] Aaby P Jensen H Gomes J Fernandes M Lisse IM. The introduction of diphtheria-tetanus-pertussis vaccine and child mortality in rural Guinea-Bissau: an observational study. Int J Epidemiol (2004) 33:374–80. 10.1093/ije/dyh005 15082643

[13] SAGE Non-Specific Effects of Vaccines Working Group. Evidence based recommendations on non-specific effects of BCG, DTP-containing and measles-containing vaccines on mortality in children under 5 years of age. Background Paper for SAGE Discussions Geneva (2014).

[14] Global Advisory Committee on Vaccine Safety. Wkly Epidemiol Rec (2004) 79:269–72 15341368

[15] Higgins JPT Soares-Weiser K Reingold A Systematic Review of the Non-Specific Effects of BCG, DTP and Measles Containing Vaccines. (2014). Available from: http://www.who.int/immunization/sage/meetings/2014/april(accessed June 1, 2014)

[16] Strategic Advisory Group of Experts on Immunization. Wkly Epidemiol Rec (2014) 89:233–5. 24466571

[17] WHO. Immunization and vaccine related implementation research advisory committee (IVIR-AC): summary of conclusions and recommendations 17–19 September 2014 meeting. Wkly Epidemiol Rec (2015) 90:1–8. 25585441

[18] Lund N Andersen A Hansen AS Jepsen FS Barbosa A Biering-Sørensen S The effect of oral polio vaccine at birth on infant mortality: a randomized trial. Clin Infect Dis (2015) 61:1504–11. 10.1093/cid/civ617 26219694 PMC4614411

[19] Andersen A Fisker AB Rodrigues A Martins C Ravn H Lund N National Immunization Campaigns with Oral Polio Vaccine (OPV) Reduce the General All-Cause Mortality Rate: An Analysis of the Effect of Campaign-OPV on Child Mortality within Seven Randomised Trials. Front. Public Health (2018) 6:13 10.3389/fpubh.2018.00013 29456992 PMC5801299

[20] Aaby P Ravn H Roth A Rodrigues A Lisse IM Diness BR Early diphtheria-tetanus-pertussis vaccination associated with higher female mortality and no difference in male mortality in a cohort of low birthweight children: an observational study within a randomised trial. Arch Dis Child (2012) 97(8):685–91. 10.1136/archdischild-2011-300646 22331681 PMC3409557

[21] Aaby P Andersen A Ravn H Zaman K. Co-administration of BCG and diphtheria-tetanus-pertussis (DTP) vaccinations may reduce infant mortality more than the WHO-schedule of BCG first and then DTP. A re-analysis of demographic surveillance data from rural Bangladesh. EBioMedicine (2017) 22:173–80. 10.1016/j.ebiom.2017.07.012 28784413 PMC5552225

[22] Kristensen I Aaby P Jensen H. Routine vaccinations and child survival: follow up study in Guinea-Bissau, West Africa. Br Med J (2000) 321:1435–8. 10.1136/bmj.321.7274.1435 11110734 PMC27544

[23] Moulton LH Rahmathullah L Halsey NA Thulasiraj RD Katz J Tielsch JM. Evaluation of non-specific effects of infant immunizations on early infant mortality in a southern Indian population. Trop Med Int Health (2005) 10:947–55. 10.1111/j.1365-3156.2005.01434.x 16185228

[24] Welega P Nielsen J Adjuik M Debpuur C Ross DA Ravn H Non-specific effects of diphtheria-tetanus-pertussis and measles vaccinations? An analysis of surveillance data from Navrongo, Ghana. Trop Med Int Health (2012) 17:1492–505. 10.1111/j.1365-3156.2012.03093.x 23006334

[25] Velema JP Alihonou EJ Gandaho T Hounye FH. Childhood mortality among users and non-users of primary health care in a rural West African community. Int J Epidemiol (1991) 20:474–9. 10.1093/ije/20.2.474 1917252

[26] Krishnan A Srivastava R Dwivedi P Ng N Byass P Pandav CS. Non-specific sex-differential effect of DTP vaccination may partially explain the excess girl child mortality in Ballabgarh, India. Trop Med Int Health (2013) 18:1329–37. 10.1111/tmi.12192 24103109

[27] Hirve S Bavdekar A Juvekar S Benn CS Nielsen J Aaby P. Non-specific and sex-differential effects of vaccinations on child survival in rural western India. Vaccine (2012) 30:7300–8. 10.1016/j.vaccine.2012.09.035 23022401

[28] Benn CS Fisker AB Jørgensen MJ Aaby P Why worry: vitamin A with DTP vaccine? Vaccine (2007) 25(5):777–9. 10.1016/j.vaccine.2006.09.044 17049684

[29] Kleinnijenhuis J Quintin J Preijers F Joosten LA Ifrim DC Saeed S Bacille Calmette-Guerin induces NOD2-dependent nonspecific protection from reinfection via epigenetic reprogramming of monocytes. Proc Natl Acad Sci U S A (2012) 109:17537–42. 10.1073/pnas.1202870109 22988082 PMC3491454

[30] Benn CS Netea MG Selin LK Aaby P A small jab – a big effect: non-specific immunomodulation by vaccines. Trends Immunol (2013) 34:431–9. 10.1016/j.it.2013.04.004 23680130

[31] Leentjens J Kox M Stokman R Gerretsen J Diavatopoulos DA van Crevel R BCG vaccination enhances the immunogenicity of subsequent influenza vaccination in healthy volunteers: a randomized, placebo-controlled pilot study. J Infect Dis (2015) 212:1930–8. 10.1093/infdis/jiv332 26071565

[32] Aaby P Garly ML Nielsen J Ravn H Martins C Balé C Increased female-male mortality ratio associated with inactivated polio and diphtheria-tetanus-pertussis vaccines: observations from vaccination trials in Guinea-Bissau. Pediatr Infect Dis J (2007) 26:247–52. 17484223 10.1097/01.inf.0000256735.05098.01

[33] Garly ML Jensen H Martins CL Balé C Balde MA Lisse IM Hepatitis B vaccination associated with higher female than male mortality in Guinea-Bissau: an observational study. Pediatr Infect Dis J (2004) 23:1086–92. 15626943

[34] Fisker AB Biering-Sørensen S Lund N Djana A Rodrigues A Martins CL Contrasting female-male mortality ratios after routine vaccinations with pentavalent versus measles and yellow fever vaccine. A cohort study from Guinea-Bissau. Vaccine (2016) 34(38):4551–7. 10.1016/j.vaccine.2016.07.034 27475473

[35] Klein SL Shann F Moss WJ Benn CS Aaby P RTS,S malaria vaccine and increased mortality in girls. MBio (2016) 7(2):e00514–6. 10.1128/mBio.00514-16 27118593 PMC4850267

[36] Sørup S Benn CS Poulsen A Krause T Aaby P Ravn H. Live vaccine against measles, mumps, and rubella and the risk of hospital admissions for nontargeted infections. JAMA (2014) 311:826–35. 10.1001/jama.2014.470 24570246

[37] Bardenheier BH McNeil MM Wodi AP McNicholl JM DeStefano F Risk of nontargeted infectious disease hopsitalizations among US children following inactivated and live vaccines, 2005–2014. Clin Infect Dis (2017) 65(5):729–37. 10.1093/cid/cix44 28481979 PMC5879781

