# Supplementary material for: Evidence of Increase in Mortality After the Introduction of Diphtheria–Tetanus–Pertussis Vaccine to Children Aged 6–35 Months in Guinea-Bissau: A Time for Reflection?
Source: Front Public Health. 2018 Mar 19;6:79. doi: 10.3389/fpubh.2018.00079 (PMC5868131; doi:10.3389/fpubh.2018.00079)
Supplement: Supplementary file 1 [file data_sheet_1.docx]

**Supplementary Table 1. Mortality rate per 100 person-years (deaths/person-years) according to most recent vaccination(s) for children aged 6-35 months.**

| **Children who participated in community weighing sessions** | **Mortality per 100 person-years (deaths/person-years)** |
| --- | --- |
| **Latest vaccine(s)** |  |
| OPV | 1.7 (2/119.2) |
| OPV, MV | 5.0 (1/20.1) |
| MV | 4.3 (7/164.5) |
| DTP | 3.1 (5/162.8) |
| DTP, OPV | 8.0 (23/288.3) |
| None | 5.1 (4/78.2) |
| Total | 5.0 (42/833.1) |

Notes: In the analyses of most recent vaccine, children who had received BCG or MV together with DTP were excluded.

**Supplementary table 2. Median age of vaccination and coverage at 3 years of age for BCG, DTP, OPV, and MV**

| **Median age in days (N vaccines)** | Coverage at 3 years | Median age at vaccination (days) |
| --- | --- | --- |
| BCG | 4% | 357 |
| DTP1 | 82% | 633 |
| DTP2 | 55% | 715 |
| DTP3 | 38% | 772 |
| OPV1 | 84% | 614 |
| OPV2 | 65% | 657 |
| OPV3 | 49% | 718 |
| MV1 | 82% | 348 |
| MV2 | 22% | 851 |

**Supplementary table 3. Meta-analysis of studies of the introduction of DTP and OPV in Guinea-Bissau**

| Location (reference) | Period | Age group | Hazard ratio (95% CI) for DTP-vaccinated versus DTP-unvaccinated | | |
| --- | --- | --- | --- | --- | --- |
|  |  |  | All | Girls | Boys |
| Urban Bissau (5) | 1981-1984 | 3-5 months | 5.00 (1.53-16.3) | 9.98 (0.81-123.0) | 3.93 (1.01-15.3) |
| Rural Guinea-Bissau (12) | 1984-1987 | 2-8 months followed for 6 months | 1.92 (1.04-3.52) | 2.34 (1.04-3.57) | 1.56 (0.70-3.48) |
| Urban Bissau  (present paper) | 1981-1984 | 6-35 months | 1.89 (1.00-3.55) | 2.76 (1.07-7.07) | 1.34 (0.56-3.22) |
|  |  |  |  |  |  |
| Combined |  |  | 2.14 (1.42-3.23) | 2.60 (1.57-4.32) | 1.71 (0.99-2.93) |
